# DNA bridging and looping by HMO1 provides a mechanism for stabilizing nucleosome-free chromatin

**DOI:** 10.1093/nar/gku635

**Published:** 2014-07-24

**Authors:** Divakaran Murugesapillai, Micah J. McCauley, Ran Huo, Molly H. Nelson Holte, Armen Stepanyants, L. James Maher, Nathan E. Israeloff, Mark C. Williams

**Affiliations:** 1 Department of Physics, Northeastern University, Boston, MA 02115, USA; 2 Department of Biochemistry and Molecular Biology, Mayo Clinic College of Medicine, Rochester, MN 55905, USA

## Abstract

The regulation of chromatin structure in eukaryotic cells involves abundant architectural factors such as high mobility group B (HMGB) proteins. It is not understood how these factors control the interplay between genome accessibility and compaction. *In vivo*, HMO1 binds the promoter and coding regions of most ribosomal RNA genes, facilitating transcription and possibly stabilizing chromatin in the absence of histones. To understand how HMO1 performs these functions, we combine single molecule stretching and atomic force microscopy (AFM). By stretching HMO1-bound DNA, we demonstrate a hierarchical organization of interactions, in which HMO1 initially compacts DNA on a timescale of seconds, followed by bridge formation and stabilization of DNA loops on a timescale of minutes. AFM experiments demonstrate DNA bridging between strands as well as looping by HMO1. Our results support a model in which HMO1 maintains the stability of nucleosome-free chromatin regions by forming complex and dynamic DNA structures mediated by protein–protein interactions.

## INTRODUCTION

The genomic DNA of eukaryotic cells is tightly packaged into chromatin. Various proteins can alter chromatin structure and regulate gene expression by facilitating access to packaged DNA. These changes in chromatin structure pave the way for eventual recruitment of proteins such as RNA polymerase leading to gene transcription. Abundant high mobility group B (HMGB) nuclear proteins can be DNA sequence-specific or -nonspecific, with multiple potential functions in DNA bending, chromatin remodeling, DNA repair and cellular signaling (1–4). Sequence non-specific HMGB protein functions remain mysterious, though it has been proposed that such proteins enhance the apparent flexibility of naked DNA, increase the accessibility of chromatin and serve as chaperones for transcription factors (5–7). The cellular concentration of sequence-nonspecific HMGB proteins is estimated to be about one-tenth the concentration of nucleosomes (8).

HMO1 is a *Saccharomyces cerevisiae* (yeast) HMGB protein essential for normal cell growth (9). HMO1 proteins contain a single canonical HMG box motif (Box B) and a second HMG-like box (Box A) that has been suggested to act as a dimerization domain (10,11). Interestingly, the 25 kDa HMO1 protein accumulates on ribosomal RNA genes in the nucleolus region of the nucleus (11), with approximately 10^4^ molecules per cell (10). *In vivo* functions attributed to HMO1 include maintenance of novel chromatin structure (12,13) and facilitation of ribosomal RNA transcription (9). Remarkably, HMO1 binding to active 35S ribosomal DNA (rDNA) prevents chromosome fragility in the absence of nucleosomes (12–16). This HMO1-bound histone-free chromatin is observed over ∼70% of ribosomal RNA genes (11,14,15,17–19). Thus, characterizing the organization of histone-free DNA in the presence of HMO1 is necessary to understand the most transcriptionally active chromatin in growing yeast cells.

Similar to yeast HMO1, human upstream binding factor (UBF) is also involved in stimulating rDNA transcription by Pol I and has other functional similarities (9,11). Both HMO1 and UBF are localized along the entire Pol I transcribed region of rRNA genes (11,15,17,18). Further, the UBF dimerization module can replace HMO1 Box A and preserve HMO1 function (11). Based on these *in vivo* studies, one would predict that dimerization of HMO1 through Box A is essential for its architectural function. Such dimerization, in turn, would be required for bridging and looping of DNA molecules. This compaction may characterize the nucleosome-free chromatin on rRNA genes (13).

Here we measure HMO1–DNA interactions and characterize mechanisms by which HMO1 protein may alter DNA architecture. Our results directly demonstrate DNA compaction, bridging and looping by HMO1 *in vitro*. These studies suggest a mechanism for stabilization of nucleosome-free chromatin across ribosomal RNA genes *in vivo* (13).

## MATERIALS AND METHODS

### Expression and purification of HMO1 protein

Plasmid pJ1870 encoding yeast HMO1 cloned in-frame with an N-terminal His_6_ tag in expression vector pTEV derived from pET15b (Novagen) was transformed into *Escherichia coli* BL21(DE3) (Agilent Technologies), and grown in 250-ml LB culture at 37°C with shaking until the culture reached a cell density corresponding to an OD_600_ of 0.6. Isopropyl β-D-1-thiogalactopyranoside (IPTG) was added to a final concentration of 1 mM and cells were grown at 37°C overnight with shaking, pelleted by centrifugation at 6000 g, and the cell pellet was then resuspended in 10 ml binding buffer (50 mM NaPO_4_, 300 mM NaCl, pH 7.5) containing 10 mM phenylmethlysulfonylfluoride (PMSF) and passed five times through an Emulsiflex C-5 high-pressure homogenizer (Avestin). The lysate was clarified by centrifugation at 22 000 g for 45 min at 4°C and the supernatant recovered. His_6_-tagged protein was purified using Ni-NTA agarose resin (Qiagen) per the manufacturer's recommendations. Briefly, washed Ni-NTA agarose resin was added in a 1:4 (v:v) ratio to the lysate, gently rotated at 4°C for 1 h, then loaded onto a 1.5 × 15 cm column. Resin-bound protein was washed with 200 ml wash buffer (50 mM NaPO_4_, 300 mM NaCl, 20 mM imidazole, pH 7.5). These conditions were sufficient to release bound contaminating DNA. Protein was eluted from the resin with elution buffer (50 mM NaPO_4_, 300 mM NaCl, 250 mM imidazole, pH 7.5) collecting 2 ml fractions until no protein was detectable with Bradford reagent. Fractions containing the protein of interest were combined and reduced to 2 ml using centrifugal cartridges (Vivaspin), and proteins were dialyzed at 4°C against 1 liter buffer containing 20 mM HEPES pH 7.5, 100 mM KCl, 1 mM ethylenediaminetetraacetic acid (EDTA) and 1 mM dithiothreitol (DTT), followed by a second dialysis against the same buffer containing 5% glycerol.

Purified protein was detagged using His_6_-tagged-TEV protease followed by dialysis into binding buffer to remove residual imidazole. Detagged protein was purified from the His_6_-tag and His_6_-tagged-TEV protease by column chromatography as described above, but with elution using nine steps of increasing imidazole concentration between 5 and 250 mM. Fractions with the desired protein were combined and reduced to 2 ml by centrifugal concentration and proteins were dialyzed at 4°C against 1 liter buffer containing 20 mM HEPES pH 7.5, 100 mM KCl, 1 mM EDTA and 1 mM DTT, followed by a second dialysis against the same buffer containing 5% glycerol. Protein quality was confirmed by sodium dodecyl sulphate polyacrylamide gel electrophoresis, DNA affinity quantitated by electrophoretic gel mobility shifts assays and DNA bending confirmed by enhancement of T4 DNA ligase-mediated DNA cyclization.

### Protein–DNA sample preparation for atomic force microscopy

We use 4361 bp linearized plasmid DNA pBR322. Linearization was performed by *Pvu*II digestion followed by phenol extraction (20). The freshly cleaved mica surface was exposed to 5 mM Mg^2+^ for 20 min at ambient temperature and pressure. The surface was then rinsed with distilled water and air dried. A DNA solution of 0.11 nM was deposited and allowed to incubate for 30 min, then rinsed and dried with argon gas. In order to image the protein-bound DNA complexes, 3 nM protein was incubated with 0.11 nM linearized plasmid pBR322 DNA with 10 mM Tris–HCl (pH 8.0) and 5 mM Mg^2+^, then deposited on the mica surface, left to equilibrate for 20 min, and finally rinsed and dried with argon gas.

### Optical tweezers

To investigate and characterize HMO1–DNA interactions, we use dual beam optical tweezers with 830 nm lasers, which can sustain a force up to 300 pN. The experimental setup consists of focusing two laser beams into a 1 μm diameter spot. A bead of high refractive index compared to the surrounding medium will be attracted to the focal point. A second bead is immobilized by a glass micropipette. A high resolution piezoelectric stage (0.15 nm resolution, Npoint) is used to extend the DNA. The force is measured from the refraction of the laser from the polystyrene bead. Once the single molecule is captured between the two beads, the solution around the DNA is exchanged by flowing in 10 times the flow cell volume of protein solution at fixed concentration followed by thermal equilibration and DNA stretching. The buffer solution consisted of 100 mM Na^+^ and 10 mM HEPES pH 7.5. Bridging and looping effects were observed by holding DNA molecules at stretching forces <1 pN.

To quantify the binding of HMO1 to DNA and the resultant loop size, force extension curves were fitted to the extensible worm-like chain (WLC) model:

(1) \begin{equation*} b_{\rm ds} \left( F \right) = B_{\rm ds} \left[ {1 - \frac{1}{2}\left( {\frac{{k_{\rm B} T}}{{P_{\rm ds} F}}} \right)^{{\raise0.5ex{\scriptstyle 1} \kern-0.1em/\kern-0.15em \lower0.25ex{\scriptstyle 2}}} + \frac{F}{{S_{\rm ds} }}} \right] \end{equation*}

where $b_{\rm ds}$ and *F* are the measured extension and force respectively, $P_{\rm ds}$ is the persistence length, $B_{\rm ds}$ is the contour length of the DNA, measured in units of nm/bp and $S_{\rm ds}$ represents the elastic modulus measured in units of pN, which takes into account the backbone extensibility. The fitting parameters $P_{\rm ds}$, $B_{\rm ds}$, $S_{\rm ds}$, provide information about the mechanical properties of the single DNA molecule.

To estimate values of the dissociation constant, *K*_D_, and the cooperativity parameter, *ω*, the McGhee–von Hippel equation was applied, treating the DNA as a lattice of binding sites:

(2) \begin{eqnarray*}
&& \Theta = \frac{cn}{K_{\rm D}}(1-\Theta )\left [ \frac{(2\omega -1)(1-\Theta )+\Theta /n-R}{2(\omega -1)(1-\Theta )} \right ]^{n-1} \nonumber \\
&& \left [ \frac{1-(n+1)\cdot \Theta /n+R}{2(1-\Theta )} \right ]^{2}
\end{eqnarray*}

(3) \begin{equation*}
{R} = \sqrt {({1 - ({n + 1}) {\cdot} {\Theta / n})}^2 + \frac{{4\omega \Theta}}{n}({1 - \Theta})} .
\end{equation*}

Here *Θ* is the DNA fractional site occupancy, *K*_D_ is the dissociation constant, *n* is the binding site size (*n* = 26 bp), *c* is the concentration and *ω* is binding cooperativity parameter.

The persistence length is given by (21)

(4) \begin{equation*} P_{\rm ds}(\Theta ) = \frac{{P_{\rm L} {\cdot} P_{\rm D}}}{{P_{\rm L} + {\Theta} \cdot (P_{\rm D} - P_{\rm L})}}, \end{equation*}

where $P_{\rm D}^{}$ is the protein-free value of $P_{\rm ds}^{}$ and $P_{\rm L}^{}$ is the protein-saturated value of $P_{\rm ds}^{}$.

The contour length is given by

(5) \begin{equation*} B_{\rm ds}(\Theta ) = B_{\rm D} + \Theta \cdot (B_{\rm L} - B_{\rm D}), \end{equation*}

where $B_{\rm D}^{}$ is the protein-free value of $B_{\rm ds}^{}$, and $B_{\rm L}^{}$ is the protein-saturated value of $B_{\rm ds}^{}$.

The overstretching force is given by

(6) \begin{equation*} F_{\rm ov} \left( \Theta \right) = F_{\rm ov}^{\rm D} + \Theta \cdot \left( {F_{\rm ov}^{\rm L} - F_{\rm ov}^{\rm D} } \right), \end{equation*}

where $F_{\rm ov}^{\rm D}$ is the protein-free value of $F_{\rm ov}^{}$ and $F_{\rm ov}^{\rm L}$ is the protein-saturated value of $F_{\rm ov}^{}$. In each of these cases (Equations 4–6), *Θ*(*c*) is determined by fitting the parameter on the left ($P_{\rm ds}(\Theta )$,$B_{\rm ds}(\Theta )$, or $F_{\rm ov} \left( \Theta \right)$) to the functional dependence in Equations (2) and (3). We then varied *K*_D_, *ω* and the saturated value of the parameter while holding *n* constant at *n* = 26 in order to find the best fit values for the three fitting parameters that minimized *χ*^2^. *n* is held constant in order to minimize the number of variables being fit to the data, and it is estimated from previous binding site size measurements (10).

### Atomic force microscopy imaging

A Bruker Nanoscope V MultiMode 8 atomic force microscope is used with Peak-Force Tapping™ mode. In this mode, a force curve is obtained at every pixel of the image. The peak force is used as a feedback parameter in order to image topography. The sample can be scanned at lower forces and with shorter contact time, thus protecting delicate samples. For imaging in air, a silicon cantilever is used (resonance frequency = 70 kHz, spring constant = 0.4 N/m and tip radius = 2 nm). The experiments were performed at room temperature. Images are processed using Nanoscope Analysis software, which consists of subtracting the average of each line in order to remove planar artifacts. The scan range used was 1 μm × 1 μm and 2 μm × 2 μm at 512 × 512 pixels and at 1024 × 1024 pixels, respectively. To quantify the atomic force microscopy (AFM) images, DNA molecules were traced and analyzed with NCTracer, software developed by the Neurogeometry Lab at Northeastern University (22,23).

To characterize the persistence length *p* of the DNA in the absence and in the presence of HMO1, orientation differences, *θ*, for various locations along the DNA were measured as function of contour length, L, and fit to the 2D WLC model

(7) \begin{equation*}
\langle \cos ({ \theta })\rangle = e^{ - L/2p} .
\end{equation*}

## RESULTS

### HMO1 binds cooperatively to single DNA molecules

**Figure 1.**
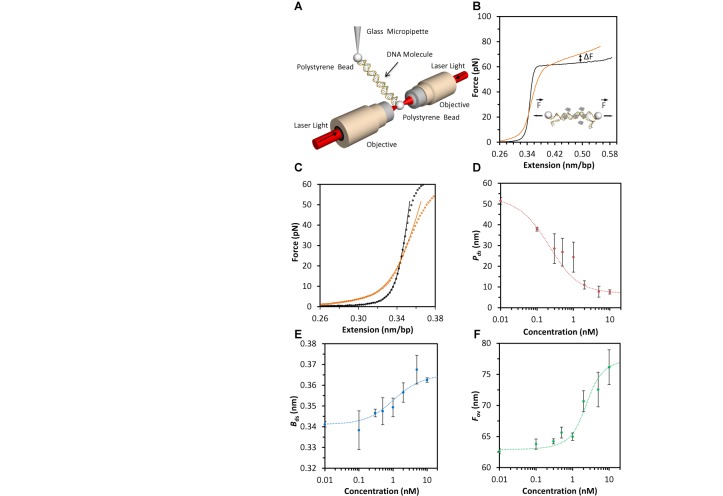
Characterization of the equilibrium binding constant and the cooperativity parameter using optical tweezers. (**A**) Schematic of optical tweezers. Phage-λ DNA (not to scale) is captured between two beads held on one side by two focused laser beams and on the other side by a micropipette. The DNA is stretched by moving the micropipette and measuring the resulting force with the optical tweezers. (**B**) The force-extension curve is obtained by stretching the DNA in the absence (black line) and in the presence of 1 nM HMO1 (orange line). The change in overstretching force in the presence of HMO1 proteins is indicated by Δ*F*_ov_ (**C**) To study the change in mechanical properties of the DNA in the presence of HMO1, data points (open symbols) in the presence (orange) and absence (black) of HMO1 are fit using the extensible WLC model (Equation 1, solid lines). (**D**) The persistence length versus concentration (symbols) is fit (dotted line) to Equation (4). (**E**) The contour length versus concentration (symbols) is fit (dotted line) to Equation (5). (**F**) The overstretching force versus concentration (symbols) is fit (dotted line) to Equation (6). The error bars indicate standard error of measurement for *N* ≥4 for all panels.

We use optical tweezers to characterize the interactions between DNA and the HMO1 protein. To do this, a single bacteriophage-λ DNA molecule of 48 500 base pairs (bp) is captured between two beads and stretched (Figure 1A). By measuring the force on the DNA molecule as it is extended and released, we obtain a force-extension curve that contains physical information about the molecule (Figure 1B, black curve). HMO1 proteins are then introduced into the fluid flow cell containing the single DNA molecule as it is held at a low force. After the DNA molecule and the HMO1 proteins have reached equilibrium, a reversible force-extension curve is obtained (Figure 1B, orange curve). In order to determine the mechanical properties of the DNA in the presence of HMO1 proteins (Figure 1C), we fit the force-extension curves up to 30 pN to the extensible WLC model (Equation 1 in Materials Methods) for several HMO1 concentrations. From these fits, we obtain three parameters: apparent DNA persistence length $P_{{\rm ds}}$, contour length $B_{{\rm ds}}$ and elastic stretch modulus $S_{\rm ds}$. We observe that the persistence length, which describes the global flexibility of the DNA–protein complex, decreases dramatically as the concentration of HMO1 is increased (Figure 1D). The dashed red line represents a fit to the cooperative McGhee–von Hippel lattice binding model (21,24) (Equations 2–4). This is a three-parameter fit in which the cooperativity parameter *ω*, the dissociation constant *K*_D_ and the saturating protein concentration are varied, while the binding site size is kept constant at *n* = 26 bp, as estimated from electrophoretic mobility shift assays (10). *ω* is a dimensionless quantity that illustrates the ease of recruiting a protein from the surrounding medium when a protein is already bound to the lattice via protein–protein interactions. We find that *K*_D_ = 2.1 ± 0.8 nM (uncertainties determined from the fit) and *ω* = 20 ± 7 from fits to the concentration dependence of $P_{\rm ds}$ (Equation 4). $B_{{\rm ds}}$ increases with concentration (Figure 1E), consistent with partial DNA unwinding and HMGB intercalation (21) and fitting these data yields *K*_D_ = 1.9 ± 0.7 nM and *ω* = 18 ± 5 (Equation 5). We also observe an increase in the overstretching force, $F_{{\rm ov}}$, with concentration, which indicates that duplex DNA is stabilized by HMO1. The concentration-dependent overstretching force is obtained by averaging the force over the extension ranging from 0.42 to 0.48 nm/bp (Figure 1B), and fits to these data yield *K*_D_ = 2.8 ± 0.6 nM and *ω* = 80 ± 15 (Equation 6). Although the latter parameter differs from the other two measurements of *ω*, the agreement between all three measurements is reasonable for independent determinations from a three-parameter fit. The fits were done keeping *n* constant, and a small change in *n* did not significantly alter the results. However, the binding site size may be force dependent, which may be reflected in the high value of *ω* observed for the overstretching force titration measurements. Overall, we obtained similar values of *K*_D_ and *ω* with the associated errors from three independent analyses. Finally we determine the weighted mean (25) and the uncertainty of the weighted mean for these three independent fits to the model, resulting in *K*_D_ = 2.3 ± 0.4 nM and *ω* = 23 ± 4. The average cluster size for proteins bound to DNA at concentrations near *K*_D_ is estimated from the McGhee–von Hippel model to be approximately two for this value of *ω*, consistent with the formation of dimers and a moderately cooperative binding protein (24).

### Force-dependent formation and disruption of loops on single DNA molecules in the presence of HMO1

**Figure 2.**
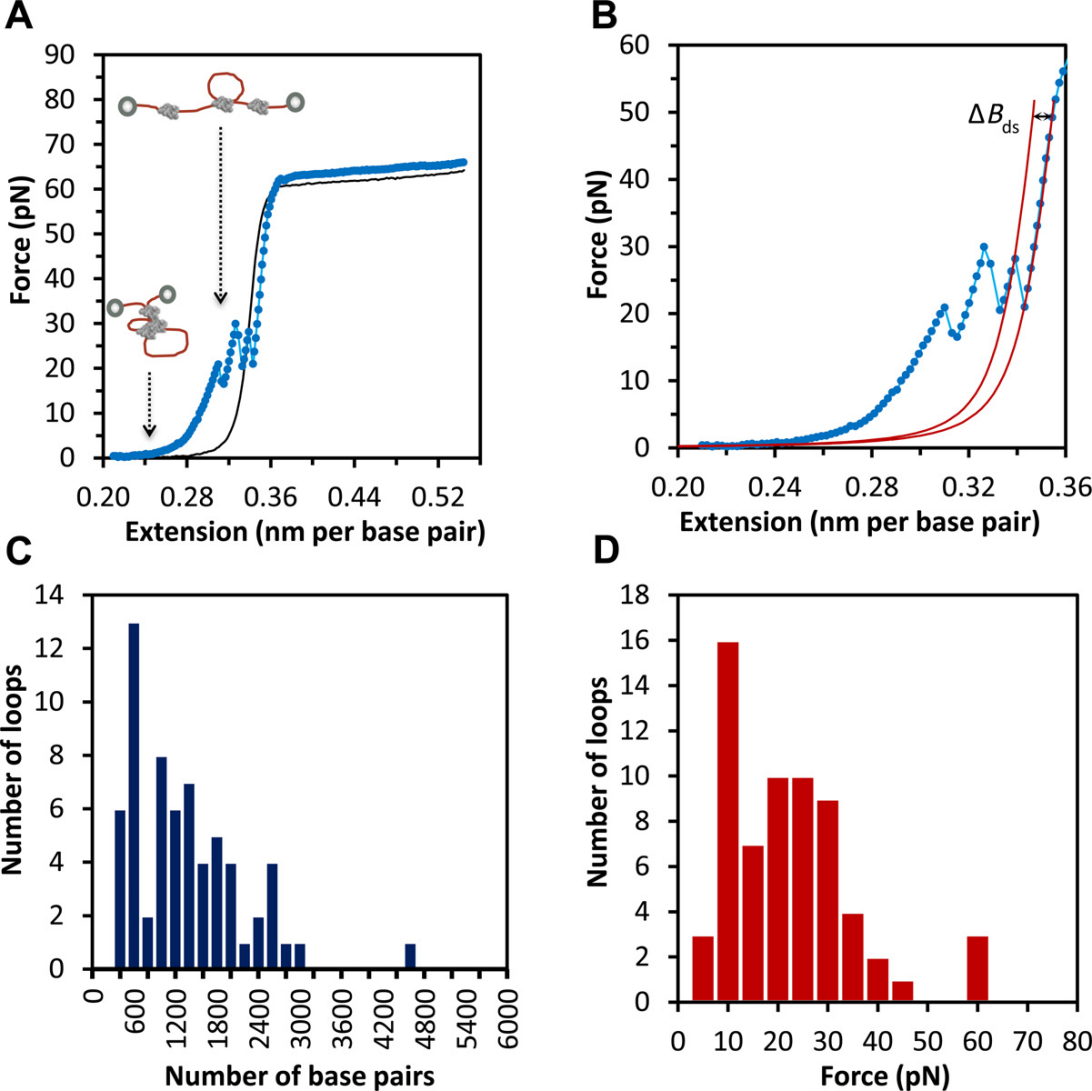
Breaking of loops formed by HMO1 proteins bound to phage-λ DNA characterized by optical tweezers. (**A**) Extension of phage-λ DNA curves are shown for phage-λ DNA in the absence (black) and presence (blue) of 0.3 nM HMO1 proteins when DNA was initially held at low forces (*F* < 1 pN). The extension curves show breaking of HMO1-bound DNA loops (observed jumps). (**B**) Extension curves of phage-λ DNA in the presence of 0.3 nM HMO1. Each jump represents an unbinding event. The loop size can be quantified by measuring the contour length change over the force jump by fitting to the WLC model (solid red lines). Δ*B*_ds_ represents the fitted length change, which is the loop size. We kept constant the value of persistence length and elastic modulus for these fits. (**C**) Histogram of loop sizes in base pairs. The most probable loop size corresponds to a range between 400 and 600 bp. (**D**) Histogram of loop breaking forces. The most probable force to break a loop is between 10 and 15 pN.

In the above experiments, when the DNA was always held at high forces, the curves were reversible and could be used to characterize equilibrium DNA binding. Similarly, when a single DNA molecule is held at low forces (*F* <1 pN) for several minutes, the subsequent force-extension curve does not change (Figure 2A, black curve). In contrast, if the molecule is held at low forces in the presence of HMO1, the force-extension curve exhibits jumps (Figure 2A, blue curve), which likely represent the non-equilibrium breaking of loops that were formed naturally due to the flexibility of DNA (26) and stabilized by HMO1 when the DNA molecule was held at low forces (*F* < 1 pN). In order to determine the size of the loops formed by HMO1-bound DNA, the change in contour length Δ*B*_ds_ was obtained by fitting every jump of the force-extension curve to the WLC model (27) (Equation 1), keeping constant the persistence length and the elastic modulus (Figure 2B) to obtain *B*_ds_ before and after the loop is broken. The loop size was determined by multiplying Δ*B*_ds_ (in nm/bp) by the number of base pairs in the DNA molecule and dividing by the DNA length per base pair, 0.34 nm. To avoid counting jumps due to noise, we did not count jumps in force that were less than 1.0 pN (our maximum force noise level in the presence of protein). A 1.0 pN jump corresponds to a loop size of 200–300 bp, depending on the force at which the jump occurs. A histogram of loop sizes for 65 unbinding events is reported in Figure 2C. When using a pulling rate of 970 nm/s, the most probable loop size corresponds to a range of 400–600 bp, the value expected for dsDNA in the absence of force (26). However, this should be considered a maximum value for most probable loop size, as the smaller loops neglected in our analysis would likely shift the distribution to smaller loop sizes, which would be expected for the lower persistence length observed in the presence of HMO1 and the fact that applied forces should also lower the loop size. The forces involved in breaking the observed loops were quantified and are reported in Figure 2D. The distribution of forces required to break HMO1-mediated DNA loops is very broad, with peak at 10 pN. However, in order to observe these loops, we had to pull the DNA at a very high rate of 970 nm/s. When pulled at rates ≤100 nm/s, the number of loop breaking events was drastically reduced. While pulling at this high rate allowed us to determine the sizes of the loops that were formed at low forces, on longer timescales these loops likely spontaneously break, suggesting a dynamic protein–DNA complex in the presence of HMO1.

### Dynamics of HMO1-induced looping and DNA compaction

**Figure 3.**
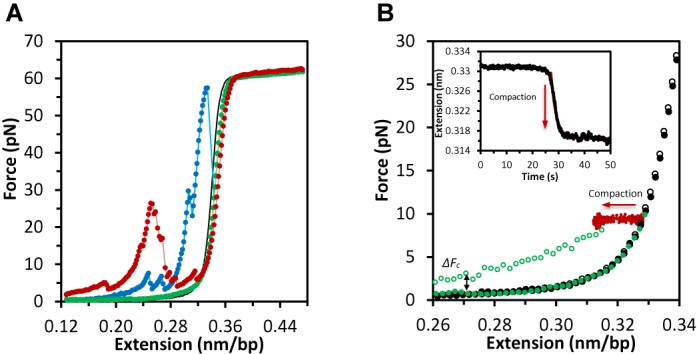
Dynamics of loop formation and compaction in the presence of HMO1. (**A**) Consecutive extension curves in the absence (black) and presence of HMO1 proteins (blue, green and red). The first extension curve (blue) shows DNA loop disruption, but the second extension curve (green) immediately after shows no DNA loops. However, after waiting for ∼5–7 min at *F* < 1 pN and then stretching a third time, loops have reformed at different locations, as shown by the red extension curve. (**B**) Compaction of a single DNA molecule by HMO1 determined by constant force measurement. The filled and open black circles show the extension and release curves in the absence of HMO1. The green filled circles show the extension of the DNA molecule before flowing the protein. This curve follows the black circles up to 10 pN, where the DNA is held and exposed to 10 nM HMO1. The red dots show the decrease in length at constant force as the DNA is compacted. The open green circles represent the release curve after the single DNA molecule has been held at constant force in the presence of HMO1 for several minutes. This curve does not overlap with the release curve in the absence of protein, showing that the DNA molecule has been compacted. The compaction force, or additional force due to protein binding in the limit of low extension, is measured to be Δ*F*_c_ = 1.7 ± 0.3 pN (averaged over five DNA molecules). The inset shows the change in extension as a function of time during compaction (black). The rate of compaction is determined by fitting to a single exponential (red line) with *k* = 0.64 ± 0.10 s^−1^ (*τ* = 1.6 ± 0.2 s).

To investigate the kinetics of loop formation and disruption, we measured consecutive cycles of extension and release curves in the presence of 0.3 nM HMO1 (Figure 3A). While the first extension curve reveals evidence of loop disruption events (blue), a subsequent extension curve (green), performed immediately after the first, does not display such jumps. This result suggests that HMO1-mediated DNA loops did not quickly reform under these conditions. However, if a 5–7-min incubation period at low forces separates the extension measurements (red), then loops of different sizes are observed to have reformed with different characteristics. The change in loop location on the force-extension curve suggests that the loop size and/or location has changed. Incubation times of <4 min did not result in significant loop formation, showing that the timescale for loop formation is several minutes. To study the compaction of a single DNA molecule by HMO1, we performed extension measurements while the DNA was held at a constant force of 10 pN using a force feedback procedure (Figure 3B). The extension and release curves in the absence of HMO1 (black) overlap. The extension of the DNA molecule in the absence of protein (solid green circles) follows the black curve up to 10 pN, at which point the DNA was held at constant force and exposed to 10 nM HMO1 protein, resulting in a gradual decrease in extension with time (red dots). The release curve (open green circles) after the single DNA molecule has been exposed to HMO1 does not overlap with the release curve for DNA only, indicating that the DNA molecule has been compacted by HMO1 at constant force. The force of compaction, determined as the force in the limit of low extensions, is measured to be Δ*F*_c_ = 1.7 ± 0.3 pN (mean ± standard error for five DNA molecules). The bead separation in the presence of HMO1 has decreased as a function of time as shown in the inset. The rate of compaction can be determined by fitting to a single exponential and a value of *k* = 0.64 ± 0.10 s^−1^ (time constant *τ* = 1.6 ± 0.2 s) is obtained. These results demonstrate that HMO1 progressively compacts DNA upon binding on a timescale of seconds. This compaction is different than the formation of large loops observed at very low force, which are likely held together at a few locations. Instead, the compaction probably represents cooperative protein binding that forms a dense protein–DNA complex.

### AFM images confirm the increase in flexibility of HMO1-bound DNA

**Figure 4.**
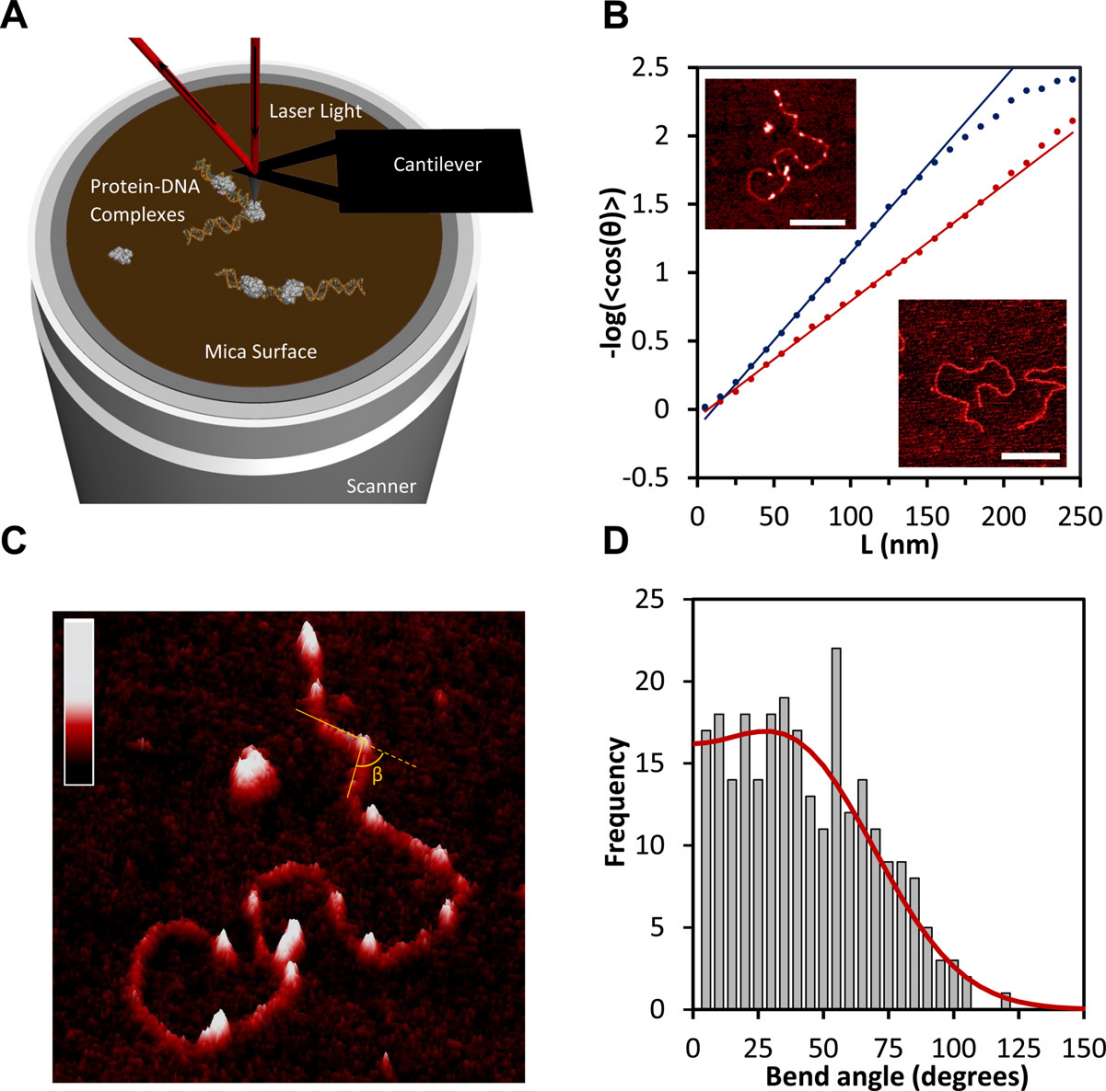
AFM images of linearized pBR322 DNA and DNA–HMO1 complexes deposited on a Mg^2+^-treated mica surface, revealing the global and local flexibility of the DNA in presence of HMO1. (**A**) Schematic of the AFM. The deflection of a laser beam is converted into a topographical imaging signal. Thus, HMO1–DNA complexes (not to scale) can be probed locally at the single molecule level. (**B**) Fits to 2D WLC model (Equation 7) allow us to determine the persistence length of DNA in the absence (red) and in the presence of HMO1 (blue). AFM images of 4361 base pair linearized plasmid DNA pBR322 in the absence of protein (lower right, scale bar 300 nm) and in the presence of protein (upper left image, white dots representing protein bound, scale bar 200 nm) are also shown. The concentration of HMO1 used is 3 nM and the concentration of DNA is 0.11 nM. **(C)** A three-dimensional representation of locally probed HMO1–DNA complexes. AFM images make it possible to resolve protein-bound sites from DNA only. Binding of the proteins is observed both at DNA termini and at sites of protein-induced DNA bends. The color bar represents the sample height ranging from 0.0 to 2.0 nm. **(D)** The distribution of HMO1-induced DNA bend angles is shown, along with its bi-Gaussian fit (red curve).

To further characterize the mechanism of flexibility enhancement and looping events detected by optical tweezers, we used AFM (Figure 4A) and determined the topography of a surface decorated by DNA and protein molecules. As a control, linear DNA molecules (0.1 nM) were first deposited on a Mg^2+^-coated mica surface and imaged in air (Figure 4B, lower right inset). To verify equilibration, a persistence length of 59 ± 2 nm for DNA in the absence of proteins was calculated by fitting to the two-dimensional WLC model (Figure 4B, red curve, Equation 7 in Methods). This result is consistent with expectations based on prior AFM measurements of DNA persistence length in air for a DNA equilibrated on a planar surface (20,28,29). The presence of 5 mM Mg^2+^ ions at very low DNA concentration did not cause dramatic changes in the measured persistence length. Linear DNA molecules (0.1 nM) were then incubated with HMO1 protein (3 nM) and the resulting protein–DNA complexes were imaged and quantified (Figure 4B). A persistence length of 39 ± 2 nm is calculated by fitting to the two-dimensional WLC model (Figure 4B, blue curve, Equation 7). The decrease in persistence length demonstrates an increase in flexibility of the DNA in the presence of HMO1, which is consistent with the trend observed with optical tweezers experiments. Here Mg^2+^ ions were used to equilibrate the HMO1–DNA complex on the mica surface. Since AFM images can resolve protein-bound locations from DNA only, we have investigated the nature of this observed global flexibility by locally probing induced DNA bend angles (Figure 4C). We find a broad distribution of bend angle (Figure 4D). A fit to a bi-Gaussian (30) (Figure 4D, red curve) yields an average bend angle of 38 ± 2° and a standard deviation of 33 ± 3°. The observed distribution of angles suggests that the protein makes the DNA locally more flexible, as found for other HMGB proteins (30).

### AFM images illustrate bridging and looping of DNA by HMO1

**Figure 5.**
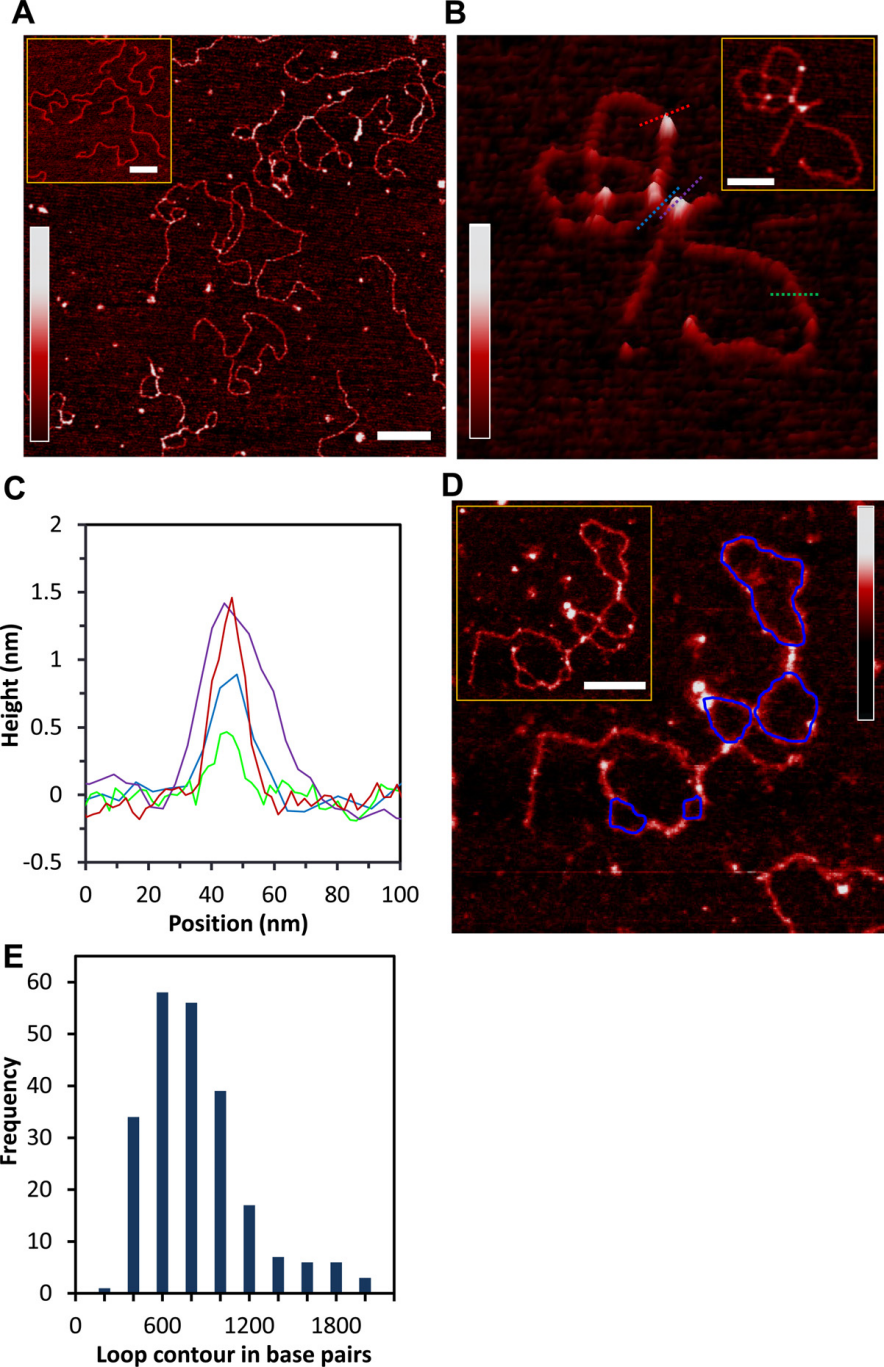
Architectural role of HMO1 in bridging, looping and compacting DNA. (**A**) Two-dimensional representation of bridges and loops mediated by HMO1 (scale bar 200 nm). We imaged 174 DNA molecules, 70% of the molecules had bridges and, on average, there were two bridges per molecule. The concentration of HMO1 used is 3 nM and the concentration of DNA is 0.11 nM. The inset shows the linearized plasmid DNA pBR322 in the absence of protein (scale bar 300 nm). (**B**) Three-dimensional representation of a looped single DNA molecule. Cross sections of DNA only, DNA with proteins bound, proteins bridging two strands of DNA and bridged DNA are shown in green, red, purple and blue, respectively. The inset represents a two-dimensional representation of locally probed HMO1–DNA complexes (scale bar 100 nm). **(C)** Graphs of the heights are shown for each cross section on the image in (B). (**D**) A two-dimensional representation of a single DNA molecule displaying multiple loops and bridges mediated by HMO1 with loops resulting from computation tracing shown in blue. Inset: Original AFM image without traces (scale bar 100 nm). (**E**) Histogram of loop sizes mediated by HMO1. The color bar in each panel represents the sample height ranging from 0.0 to 2.0 nm.

We imaged 174 HMO1–DNA complexes. HMO1 proteins bound both internally and at DNA termini, induced bends, and formed bridges and interlocked structures frequently involving DNA loops (Figure 5A, B and D). We determined that 70% of molecules had bridges, defined as two DNA strands connected by one or more proteins. On average there were two bridges per molecule. In addition to two-dimensional images, we also show a three-dimensional representation of a single molecule with HMO1-mediated loop formation (Figure 5B). Representative cross sections of a DNA molecule in the absence of proteins, including a region with proteins bound, a region with proteins bridging two strands of DNA, and two DNA strands held together by protein bridges, are shown in green, red, purple and blue, respectively (Figure 5B and C). We then quantified the sizes of the loops mediated by HMO1 (Figure 5D) by tracing each loop using NCTracer (see Materials and Methods) and directly calculating the number of base pairs along the contour. The most probable loop size was found to be in the range of 400–600 bp (31,32) (Figure 5E). Because this loop size corresponds to the size of DNA loops in solution, these results suggest that HMO1 binds to the DNA crossover regions and stabilizes stochastically created loop structures. Although one might expect the loop size to be significantly reduced in the presence of protein due to the reduction in persistence length, this result is not surprising because the effective concentration of protein in solution in the AFM experiments is lower due to the presence of competitor DNA (33). Based on the persistence length measured in the AFM experiments, 39 nm (4B), the fractional binding of HMO1 can be directly calculated from Equation (4) to be 0.07. Therefore, for a protein binding site size of 26 bp, this means that proteins are only bound on average every 400 bp, severely limiting the protein's ability to reduce the loop size, but still allowing formation of loops and bridges.

## DISCUSSION

It has been shown that sequence-nonspecific HMGB proteins compact DNA by bending the helix with a reduction of apparent DNA persistence length, while elongating DNA by intercalating between its base pairs (27,34). Not surprisingly, both of these effects are observed here (Figure 1D and E). HMGB-induced DNA compaction in the absence of looping may reflect flexible hinge motion, transient rigid bends or some combination of these effects at protein binding sites (5–7,35–37). When DNA looping is suppressed by high forces, we observe that HMO1 reduces the apparent DNA persistence length. This enhanced flexibility may play a role in gene activation and in the compaction of histone-free chromatin regions. Our observation of DNA shortening under a constant force of 10 pN in the presence of HMO1 indicates active DNA compaction on a timescale of *τ* = 1.6 s, which may facilitate the protection of nucleosome-free chromatin regions observed *in vivo* (13). The measured stretching curves were used to characterize the HMO1 concentration-dependence of the DNA persistence length, contour length and overstretching force. The resulting binding affinity is similar to that previously measured for a double box HMGB protein (27). Therefore, it is likely that the second HMG-like box contributes to the overall DNA binding affinity of HMO1. We also observe moderately cooperative binding of HMO1 to DNA, with a cooperativity parameter of *ω* = 23 ± 4. This extent of cooperative binding suggests that near *K*_D_, the average cluster size of proteins bound to DNA is approximately two (24), consistent with dimerization of HMO1 upon binding to DNA, as observed *in vivo* (11).

HMO1 was observed to stabilize DNA loops when DNA was held at a force below 1 pN. Under these conditions, we observe an increase in force at extensions less than the DNA contour length, followed by a sudden decrease in force as the loops break during extension (Figure 3A). In a subsequent stretching cycle (Figure 3A, green curve), we do not observe loops unless additional time is provided for interactions (Figure 3A, red curve). The timescale of seconds for compaction and minutes for loop formation suggests a hierarchical reorganization process of bending and compaction followed by looping. This result reveals the importance of protein–protein interactions, in addition to DNA bending, at the protein binding site.

The looped DNA structures formed in the presence of HMO1 are only stable on short timescales, as pulling at a slower rate of 100 nm/s reduced the number of observed loops by a factor of 10. Because molecular motors such as RNA polymerase can exert forces on the order of 10 pN (38–40), it should be possible for RNA polymerase to very easily move through DNA compacted by HMO1 as long as the rate of movement is 100 nm/s or less. The transcription rate of the yeast RNA polymerase on DNA only was determined in single molecule experiments to be 12.2 ± 4.5 nucleotides per second (41), which corresponds to ∼4 nm/s. Thus, while the loops formed by HMO1 locally compact DNA, RNA polymerase should be able to move through these structures without being significantly slowed down. In contrast, the force required to completely unravel nucleosomes is much higher, ∼15–25 pN (42,43), and these structures on their own are quite stable, so it should be much more difficult for RNA polymerase to disrupt nucleosomes. While optical tweezers allow loop formation to be measured only on a single molecule, the AFM images demonstrate that HMO1 stabilizes complex, interstrand DNA structures as well as loops.

In prokaryotic and archaeal cells, the organization of genomic DNA is also mediated by several sequence-nonspecific DNA binding proteins. In prokaryotic cells, abundant proteins such as HU and H-NS presumably organize the genomic DNA into an unknown nucleoid structure through DNA bending and bridging. These proteins also facilitate transcription by introducing DNA flexibility and tight bending and looping (35,36,44,45). HMO1 compacts and bridges DNA, reminiscent of the properties of *E. coli* H-NS protein (44,45). Archaeal chromatin is shaped by proteins such as Alba, which has also been shown to loop DNA (46). Analogous to the bacterial genome, the human mitochondrial genome and yeast mitochondrial genome are shaped by architectural proteins such as TFAM and Abf2p, respectively (47). Although TFAM and Abf2p both contain two HMG boxes, looping has not been observed for Abf2p (48), whereas some studies of TFAM have observed looping (49,50) and another study has suggested that its compaction mode does not involve any looping (51). Here we show by combining optical tweezers and AFM studies that the eukaryotic nuclear HMGB protein HMO1 is also capable of modulating genomic DNA structure. Although the detailed structures formed *in vivo* are likely to be more complex than those observed here, our results suggest that HMO1 shapes the specialized chromatin of highly-transcribed yeast ribosomal RNA genes using a hierarchical organization of interactions that combines DNA compaction, bridging and looping.
